# Dr. Ashok Jain

**Published:** 2010

**Authors:** N Tandon

**Affiliations:** Hon. Secretary Gwalior City Branch, Gwalior, MP, India

**Figure:**
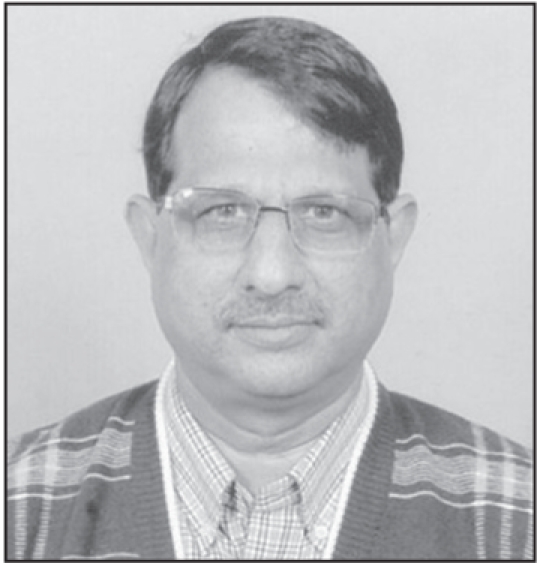
26^th^ November 1954 – 9^th^ May 2010

Dr. Ashok Jain joined G.R. Medical College, Gwalior in 1973 and completed his MBBS., and D.A. in 1981. During his life time, he started his career as Anaesthesiologist in Cancer Hospital and Research Center, Gwalior. Later on he left the job and became first Full Time anaesthesiologist in private sector of Gwalior. He was one of active member of I.S.A. and having dynamic personality.

He was also first anaesthesiologist who started Pain – Clinic at Gwalior. Since last two year, he was suffering from undiagnosed disease of spine.

He survived by his wife and two sons, elder one is an engineer and working in U.S.A., while younger is doing B.E. from Indore.

May his soul rest in peace

Gwalior

Date: 15-05-2010

